# Immune-related genes are differentially associated with negative symptoms subdomains in patients with schizophrenia

**DOI:** 10.1192/j.eurpsy.2022.732

**Published:** 2022-09-01

**Authors:** V. Golimbet, T. Lezheiko, G. Korovaitseva, N. Kolesina, V. Plakunova, V. Mikhailova

**Affiliations:** Mental Health Research Center, Clinical Genetics Laboratory, Moscow, Russian Federation

**Keywords:** immune-related genes, negative symptoms, schizophrénia

## Abstract

**Introduction:**

Negative symptoms (NS) are a core feature of schizophrenia. NS show heterogeneity proven by factor analysis, which revealed two distinct negative symptoms subdomains: diminished expression (DE) and avolition/apathy (AA) (Marder et al. 2017, Fleischhacker et al. 2019). Some studies showed the different effects of these subdomains on clinical features of schizophrenia that suggest different pathophysiological mechanisms for their development (Stanculete 2021). It has been also shown that the levels of peripheral interleukins (IL) specifically correlate with NS (Enache et al. 2021), in particular, increased IL levels were determined in patients with deficit syndrome compared to non-deficit schizophrenia (Goldsmith et al. 2018).

**Objectives:**

To search for the association of genes for IL-4, IL-6, IL-10 and C-reactive protein (CRP) with NS subdomains.

**Methods:**

The total sample included 551 patients (women 51,4%, aged 18-72 years) with ICD-10 diagnosis of schizophrenia. NS were assessed by PANSS. PANSS-derived factors include AA (items N2, N4, G16) and DE (N1, N3, N6, G5, G7, G13). Genotyping was performed for the following polymorphisms: C-589T IL-4, C-174G IL-6, C-592A IL-10, G-1082A IL-10, CRP (rs2794521).

**Results:**

There are effects of C-592A IL-10 (p=0.017) and G-1082A IL-10 (p=0.012) on AA subdomain. Post-hoc Bonferroni corrected comparisons show that carriers of the haplotype AA (C-592A)- AA (G-1082A) have the highest AA score. A significant effect of CRP (rs2794521) on AA is identified (p=0.007). There is a trend towards the association of C-589T IL-4 and C-174G IL-6 with AA. No association of these polymorphisms with DE was found.

**Conclusions:**

AA and DE may have different genetic background.

**Disclosure:**

No significant relationships.

